# A fast and robust protocol for metataxonomic analysis using RNAseq data

**DOI:** 10.1186/s40168-016-0219-5

**Published:** 2017-01-19

**Authors:** Jeremy W. Cox, Richard A. Ballweg, Diana H. Taft, Prakash Velayutham, David B. Haslam, Aleksey Porollo

**Affiliations:** 1 Department of Electrical Engineering and Computing Systems, University of Cincinnati, 2901 Woodside Drive, Cincinnati, OH 45221 USA; 2 The Center for Autoimmune Genomics and Etiology, Cincinnati Children’s Hospital Medical Center, 3333 Burnet Avenue, MLC 15012, Cincinnati, OH 45229-3039 USA; 3 Division of Biomedical Informatics, Cincinnati Children’s Hospital Medical Center, 3333 Burnet Avenue, Cincinnati, OH 45229 USA; 4 Division of Infectious Diseases, Cincinnati Children’s Hospital Medical Center, 3333 Burnet Avenue, Cincinnati, OH 45229 USA

**Keywords:** Microbiome, Metagenome, Metatranscriptome, Metataxonomics, RNAseq, Assembly of shotgun reads, Altered Schaedler flora

## Abstract

**Background:**

Metagenomics is a rapidly emerging field aimed to analyze microbial diversity and dynamics by studying the genomic content of the microbiota. Metataxonomics tools analyze high-throughput sequencing data, primarily from 16S rRNA gene sequencing and DNAseq, to identify microorganisms and viruses within a complex mixture. With the growing demand for analysis of the functional microbiome, metatranscriptome studies attract more interest. To make metatranscriptomic data sufficient for metataxonomics, new analytical workflows are needed to deal with sparse and taxonomically less informative sequencing data.

**Results:**

We present a new protocol, IMSA+A, for accurate taxonomy classification based on metatranscriptome data of any read length that can efficiently and robustly identify bacteria, fungi, and viruses in the same sample. The new protocol improves accuracy by using a conservative reference database, employing a new counting scheme, and by assembling shotgun reads. Assembly also reduces analysis runtime. Simulated data were utilized to evaluate the protocol by permuting common experimental variables. When applied to the real metatranscriptome data for mouse intestines colonized by ASF, the protocol showed superior performance in detection of the microorganisms compared to the existing metataxonomics tools. IMSA+A is available at https://github.com/JeremyCoxBMI/IMSA-A.

**Conclusions:**

The developed protocol addresses the need for taxonomy classification from RNAseq data. Previously not utilized, i.e., unmapped to a reference genome, RNAseq reads can now be used to gather taxonomic information about the microbiota present in a biological sample without conducting additional sequencing. Any metatranscriptome pipeline that includes assembly of reads can add this analysis with minimal additional cost of compute time. The new protocol also creates an opportunity to revisit old metatranscriptome data, where taxonomic content may be important but was not analyzed.

**Electronic supplementary material:**

The online version of this article (doi:10.1186/s40168-016-0219-5) contains supplementary material, which is available to authorized users.

## Background

Most naturally occurring higher organisms host microbiota. The importance of a microbiome in human health is recognized by the National Institutes of Health (NIH) via support of the Human Microbiome Project in 2007 (https://commonfund.nih.gov/hmp/), which resulted in >500 peer-reviewed publications by the project participants as of February 2016. Metagenomics is a rapidly emerging field aimed to analyze microbial diversity and dynamics by studying the microbiome (genomic content of the microbiota). Advantages in high-throughput deep sequencing enabled focused studies of microbiomes in different organisms and environmental niches. Metataxonomics tools analyze sequencing data to identify microorganisms and viruses from complex mixtures. These tools can be divided into two primary categories based on the data they process for identifying microorganisms: short marker sequencing (e.g., 16S and 18S/ITS rRNA genes for bacteria and fungi, respectively) and shotgun DNA sequencing (DNAseq). However, identification of microorganisms and understanding of their role in the host health and pathogenesis pose challenges to the bioinformatics community. The major challenges for metataxonomics are (1) processing a large volume of sequence data efficiently, (2) dealing with ambiguous information, when the same sequence matches to multiple species, and (3) classifying with resolution below the genus clade. For example, in the DNAseq analysis, sequences may align to multiple taxa, possibly in different clades [1–3]. In 16S metagenomic analysis, a sequence is mapped to an operational taxonomical unit (OTU), which represents a cluster of organisms rather than a specific organism [4].

A fundamental step in taxonomy classification is to count taxa based on the shotgun read alignments to the metagenome. Metataxonomics tools employ various strategies to produce better counts. IMSA [5] and PathSeq [6] count the number of significant sequence alignments at various levels, to species, genus, and family. Clinical Pathoscope [7, 8] and MetaGeniE [9] follow the same approach, but add an error-correcting schema. MEGAN only counts a read if the all alignments for the read unanimously agree on the taxon. Following the Lowest common ancestor (LCA) concept, MEGAN assigns the read to the lowest taxonomic category, where there is an agreement [2, 10]. MEGAN CE [11] recommends DIAMOND [12], a high-throughput algorithm that aligns shotgun reads to protein sequences. Kraken [3] determines LCA by looking up all subsequence *k*-mers in a prebuilt classification table. MetaPhlAn2 ignores the sequences that do not match to the precomputed list of genes—taxonomic markers [13, 14].

Metataxonomics programs typically have several restrictions on the data they are designed to work with. Tools with a medical inclination frequently narrow their search by the implicit assumption that there is a single microorganism causing disease (PathSeq [6], Clinical Pathoscope [7, 8], RINS [15], SURPI [16]). Such tools are less effective when studying diverse microbial communities. Moreover, a majority of published metataxonomics frameworks are tested with bacteria and/or viruses (e.g., GOTTCHA [17], VirusFinder [18], VirusSeq [19]), excluding other microorganisms like protists, algae, and fungi. Limiting the taxonomy identification to one kingdom may lead to an incomplete understanding of the studied microbiome, its interactions, and functional landscape. Moreover, the appreciation of fungal microbiome is rising [20]. Indeed, in a recent study of the oral human mycobiome, 60 nonpathogenic fungal genera were identified that are considered to be environmental in nature [21]. Typically, 100 bases or longer reads are used for testing metataxonomics tools [3, 6, 17, 22, 23], thus making their applicability to shorter reads uncertain. Lastly, though detection of microbial DNA likely translates to the presence of microorganisms, it cannot inform about the viability and functional states (e.g., metabolism) of these populations. The reader may refer to Additional file 1 “Survey of Metataxonomic Tools” for further details on existing tools.

Ribosomal depleted shotgun RNA sequencing (RNAseq) is a high-throughput sequencing technique that enables the analysis of transcriptomic landscapes of the microbiome [24–27]. The RNAseq reads assembly improves metatranscriptome functional annotation [28]. There is an opportunity to use existing RNAseq data for metataxonomics. If possible, using the same RNAseq data for both metatranscriptome functional analysis and taxonomy classification would be an efficient alternative to the DNAseq-based approach.

**Fig. 1:**
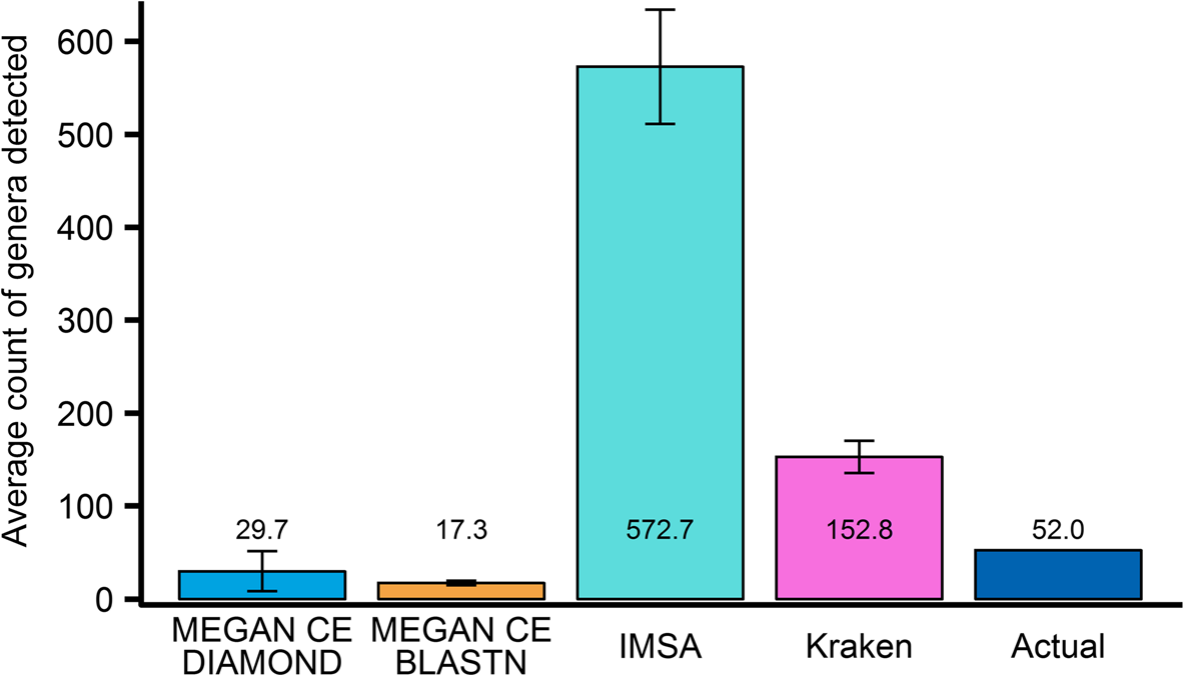
Comparison of the selected metataxonomics workflows on detection of genera within a set of simulated datasets (Table 1). IMSA and Kraken identify too many taxa. Both versions of MEGAN CE find too few taxa, most likely due to the weighted LCA that filters out noise, which also filters out weak signal of organisms present

An RNAseq-based metataxonomics faces new challenges. Our brief survey on adapting DNAseq-based taxonomy classification tools to the analysis of RNAseq shotgun reads, both simulated and real data, showed that they yield impractical results (see Fig. 1 and Additional file 1 “Performance on Real Data”). RNAseq data is distinctly different from DNAseq data. Coding regions have higher conservation across species or can be result of the horizontal gene transfer. Hence, RNAseq reads are more likely to be ambiguous regarding their origins. Furthermore, the more informative, less ambiguous regions of metatranscriptome may not be expressed under given conditions. Consequently, the taxonomy classification task with RNAseq is more difficult than that with DNAseq.

This work presents a reliable lightweight protocol that extracts taxonomic information from the RNAseq data with unknown microbial community composition, which may be compounded by abundant host reads. The new RNAseq-based metataxonomics protocol, dubbed IMSA+A, incorporates IMSA [5], transcript reads assemblers (Oases [29] and Inchworm/Trinity [30]), and a modified IMSA counting scheme for taxonomy assignments. Several simulation experiments were conducted permuting related key parameters to validate the protocol and to identify the limits of its applicability. The efficacy of IMSA+A was demonstrated using real experimental data. Several key sources of noise were identified and addressed by the protocol: the quality of the reference database, short read sequences, and taxonomy counting methodology. A conservative database, de novo read assembly, and a modified counting method were incorporated into the protocol to improve the results of metataxonomic analysis.

## Methods

### Reference sequence databases

Bacterial, fungal, and viral genomes and the corresponding transcriptomes as of March 1, 2015, were taken from the NCBI Genomes database [31]. To increase fungal representation in the reference database, additional select genomes and transcriptomes available as of June 1, 2015, were retrieved from FungiDB.org [32, 33]. For simplicity, members of *Stramenopiles*, sometimes called pseudo-fungi, were included in the database as members of the fungal kingdom. The retrieved genomes were combined to make a custom reference genome database, while transcriptomes were used to generate simulated datasets (see below). This custom database was used by IMSA, IMSA+A, and MEGAN CE BLASTN pipelines. Also, the complete NCBI RefSeq database (January 10, 2016) [34] was used as an alternative reference database when testing IMSA+A.

Our Kraken database was constructed by combining the standard Kraken database (generated by its utility) with additional complete genome sequences of microorganisms, sourced from Genbank. The database consists of 19,196 organisms in total, including 171 fungi, 3350 bacteria, 15448 viruses, and 227 others (primarily viridiplantae, metazoa, protists, and artificial sequences).

DIAMOND used the NCBI NR database as of October 4, 2016.

### Accuracy measures

To evaluate performance of our protocol, true positive rate (TPR) and false discovery rate (FDR) were defined as follows:

1 $$ \mathrm{T}\mathrm{P}\mathrm{R}=\mathrm{T}\mathrm{P}/\left(\mathrm{T}\mathrm{P}+\mathrm{F}\mathrm{N}\right) $$

2 $$ \mathrm{F}\mathrm{D}\mathrm{R}=\mathrm{F}\mathrm{P}/\left(\mathrm{T}\mathrm{P}+\mathrm{F}\mathrm{P}\right) $$

where TP is the number of correctly identified taxa (true positive), FP—the number of taxa wrongly predicted to be in the dataset (false positive), FN—the number of taxa present but not identified (false negative). Other accuracy measures are not applicable as they require true negatives (TN), which are not defined in the evaluation sets, and the protocol is not intended to predict them. Desired optimal classification performance would be TPR > 0.90 and FDR < 0.10.

### Statistics

Kruskal-Wallis test was used to evaluate the performance difference (TPR, FDR) between groups. The significance level used was *α* = 0.05.

### Simulated datasets

Simulated sequencing data were generated using Grinder [35]. Uniform random distributions, simulated by seeded Mersenne Twister [36], were used to select randomly (1) species (bacteria, fungi) or strains (viruses) from combined transcriptomes databases and (2) genes to represent an organism in simulation. The number of species and percent genes selected were chosen separately for each kingdom. In some cases, species selection was held constant to control this variable between simulations. Real gene expression is expected to vary. Since this cannot be readily defined, genes were selected at random. Each species was given an equal share of the sequencing depth allotted to each kingdom, and an equal share of that species depth was allotted to the randomly chosen genes. Thus, coverage varies between kingdoms and between organisms within a kingdom. Based on these inputs, Grinder then generated the simulated RNAseq shotgun reads in a unidirectional mode. Twenty-eight total datasets were generated representing various conditions used to evaluate the protocol.

To account for variable-relative abundance and gene expression, simulation incorporated a random relative abundance and random gene expression. Relative abundance was determined once per organism using a random uniform distribution from 1 to 20. Gene expression was randomized using the same distribution as Flux Simulator [37], which was used to randomly generate values within a range of 1 to 1000 relative units of expression. After normalization, the ultimate result is a maximum possible ratio of 1000:1 in FPKM scores for genes from the same organism (see details in Additional file 1 “Simulated Gene Expression”). Because each kingdom’s reads were simulated separately, relative abundance was subsequently impacted by the choice of the proportion of reads allocated to each kingdom.

### Transcript assemblers

The purpose of assembly in our protocol is to reconstruct putative genes thereby improving the taxonomy classification performance and reducing the computational burden of sequence alignments since millions of shotgun reads assemble into thousands of contigs. Several assemblers were recently evaluated, measuring their performance with metatranscriptome data [28]. Of these, two transcriptome assemblers, Oases [29] and Inchworm/Trinity [30], were chosen to be used in the IMSA+A protocol. Inchworm is a simple, fast, multi-threaded, de novo transcriptome assembler. It is conservative by extending reads only when there is an exact *k*-mer match. Oases operates similarly to Inchworm. However, Oases employs error correction schema. Oases merges multiple assemblies derived using various *k*-mers (an approach first described in [38, 39]) with a topological analysis for transcriptome-specific contigs corrections [26], including the elimination of cross-gene assemblies.

### Improved IMSA counting scheme

The original IMSA workflow includes (1) subtraction of host sequences from the shotgun reads (with a number of customizable parameters), (2) alignment of the remaining reads to the metagenome reference database using the megaBLAST algorithm [40], and (3) counting the number of BLAST hits to conduct taxonomy assignment. IMSA generates count reports at the species, genus, family, and division levels. In the case of ties, the count of 1 sequence splits evenly making fractional counts. All shotgun reads are considered as independent sequences. Therefore, multiple reads representing the same genomic location contribute to the counting as multiple hits. Thus, IMSA would not report whether a resulting count is due to many ambiguous alignments (scored ≤0.5 each) or because of fewer unique alignments (scored 1 each), or a combination of these two scenarios.

Our protocol uses a modified counting scheme. It calculates the original IMSA counts, but breaks the count of each taxon into (1) the number of best matching sequences without ties (unique counts or LCA counts [2]), (2) the number of sequences matching multiple taxa (ambiguous sequences), and (3) the sum of the fractional counts yielded by ambiguous sequences. Uniqueness is calculated at every clade. For example, if a sequence aligns to two different strains of *Escherichia coli*, then the sequence is counted as one unique hit for *E. coli* at the species clade level.

**Fig. 2:**
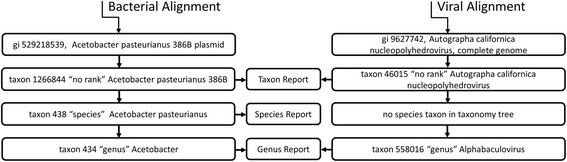
Example of processing alignments to generate reports. Alignment to a virus does not contribute to the species count, as there is no corresponding assignment in the taxonomy tree

Viruses are represented in the NCBI database with incomplete taxonomies—a distinct virus may not have a species or genus assignment. IMSA and other tools put alignment evidence into taxonomic bins. Consequently, any species- or genus-based summary of the virus counts will be incomplete and misleading. To properly report the viruses detected in the sample, they are treated with a new scheme that accounts for this peculiarity in a taxonomic classification. IMSA+A generates also report at the first taxon level (Fig. 2), which summarizes counts by the taxa identified by the BLAST alignment, without traversing the classification tree to report the alignment counts at a different clade level. The reported taxon is usually a species, a subspecies (or strain), or the designation “no rank”. No rank indicates that the taxon does not belong to a clade. In the case of plasmid sequences, IMSA will detect the organism, from where the plasmid originated, since the NCBI taxonomy tree for plasmids is structured so that each plasmid belongs to a taxon (species or strain).

Due to lack of any direct information in the database as to how taxonomically relate viruses, results for viruses were manually interpreted to compute accuracy measures. Specifically, when two supposedly related viruses (as deduced from their similar names) were identified, the virus with considerably lower count (at least tenfold) was discarded. For example, Clostridium phage PhiS63 with count 1 was detected along with Clostridium phage phiSM101 with count 53. The former was removed from the list of detected viruses.

### IMSA+A protocol

**Fig. 3:**
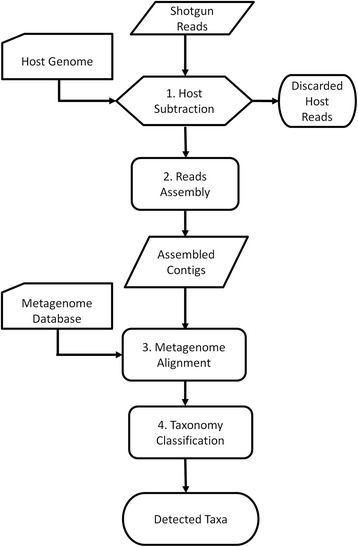
Overview of the IMSA+A protocol

The new protocol aims to determine taxonomies of the microbiota represented in the metatranscriptome data. The protocol is based on IMSA [5] and adds a read assembly step and a modified taxonomy counting scheme. Figure 3 presents a workflow of the protocol.

RNAseq data can be submitted in either the FASTA or the FASTQ formats. All reads, including those from the paired-end sequencing, will be treated as single reads per IMSA heuristic.

- Step 1. Run IMSA to subtract host reads using a host genome/transcriptome database.
- Step 2. Assemble the remaining reads.
- Step 3. Align the assembled contigs against the metagenome database.
- Step 4. Run the modified IMSA+A counter for taxonomy classification.

IMSA defines the steps of the metagenomic analysis in a high-level scripting language. To insert the assembly step into the IMSA workflow, IMSA is terminated after the host subtraction, and the last two steps are executed outside the action script. IMSA+A provides no additional options for sequence alignments beyond those offered by IMSA.

## Results

First, we demonstrate the effectiveness of the new protocol in improving classification accuracy by using a conservative reference database, a de novo assembler, and a new counting method. Additional key parameters, which usually confound classification, are permuted in simulation experiments to evaluate the protocol and identify its limitations. Then, we illustrate the performance of the developed protocol on a real RNAseq data derived from mice with a controlled microbiome, whose compositional species are not included in the reference database.

### Simulation experiments

The simulation conditions were chosen to represent difficult taxonomy classification circumstances: high number of species present from multiple kingdoms (30 bacteria, 15 fungi, and 10 viruses, as well as a variable microbial composition), high host sequencing percentage (95%) leading to low microbiome sequencing depth, and 1% sequencing error rate. Percent gene selection was chosen 25 or 100% for bacteria, 50 and 100% for fungi and viruses, respectively. Variable gene expression and relative abundance were also evaluated in an additional dataset. Sequencing depth of 70 million was chosen to reflect our real sequencing data (not presented in this work). The proportion of sequencing depth and the number of species for each kingdom were chosen to be a plausible real-world composition. About 1% of human RNA sequences (five to eight hundred thousand) remained after subtraction, and less than 0.1% of microbiome sequences were removed by subtraction step. Table 1 provides summary of the nine main simulated datasets used to evaluate the protocol.

**Table 1:** Simulated datasets used for evaluating and optimizing the IMSA+A protocol

Experiment			Parameters used to vary coverage		Other parameters controlled for this experiment												
Read length/coverage	Read length	Bacteria Coverage	Bacteria seq depth^b^	Bacteria gene selection	Bacteria species^a^	Fungi coverage	Fungi seq depth^b^	Fungi gene selection	Fungi species^a^	Virus coverage	Virus seq depth^b^	Virus gene selection	Virus strains^a^	Human coverage	Human gene coverage	Human Seq depth^b^	Human reads after subtraction
50 low	50	0.25	1.0	100%	30	1.1	2.4	50%	15	16.2	0.1	100%	10	14.19	100%	66.5	0.078
50 med	50	1.11	1.0	25%	30	1.1	2.4	50%	15	16.2	0.1	100%	10	14.19	100%	66.5	0.078
50 high	50	4.44	4.0	25%	30	1.1	2.4	50%	15	16.2	0.1	100%	10	14.19	100%	66.5	0.078
100 low	100	0.87	1.0	100%	30	2.3	2.4	50%	15	106.4	0.1	100%	10	28.31	100%	66.5	0.074
100 med	100	2.22	1.0	25%	30	2.3	2.4	50%	15	106.4	0.1	100%	10	28.31	100%	66.5	0.074
100 high	100	8.88	4.0	25%	30	2.3	2.4	50%	15	106.4	0.1	100%	10	28.31	100%	66.5	0.074
150 low	150	1.30	1.0	100%	30	3.4	2.4	50%	15	159.5	0.1	100%	10	42.46	100%	66.5	0.054
150 med	150	3.33	1.0	25%	30	3.4	2.4	50%	15	159.5	0.1	100%	10	42.46	100%	66.5	0.054
150 high	150	13.33	4.0	25%	30	3.4	2.4	50%	15	159.5	0.1	100%	10	42.46	100%	66.5	0.054

^a^Simulated organisms were the same across experiments as an experimental control

^b^Sequencing depth in millions

It should be noted that organisms chosen for all simulated datasets remain in the reference database. This enabled computation of accuracy at species level and review of different parameters that potentially may influence performance of the new protocol. However, the final section of Results presents the evaluation of the protocol on real data, when the anticipated organisms are known to be not present in the reference database. This is the ultimate test of the usability of the protocol.

### Comparison of counting schemes

The results from 36 scenarios (9 datasets × 4 workflow versions) are summarized in Additional file 2: Table S1 and Additional file 3: Table S2 for the new counting and original IMSA counting methods, respectively. The new counting scheme consistently yields a lower FDR than the original IMSA counting scheme, while maintaining the same level of TPR (Table 2).

**Table 2:** Average taxonomic classification performance by counting scheme^a^

Counting Scheme	Bacteria		Bacteria		Fungi		Fungi		Virus	
	Species level		Genus level		Species level		Genus level		First taxon level	
	TPR	FDR	TPR	FDR	TPR	FDR	TPR	FDR	TPR	FDR
Unique count >0	0.77 ± 0.12	0.45 ± 0.20	0.84 ± 0.13	0.20 ± 0.19	0.88 ± 0.11	0.62 ± 0.26	0.92 ± 0.08	0.56 ± 0.26	0.97 ± 0.10	0.07 ± 0.09
IMSA count >0	0.78 ± 0.11	0.79 ± 0.16	0.84 ± 0.12	0.58 ± 0.20	0.88 ± 0.11	0.70 ± 0.21	0.92 ± 0.08	0.64 ± 0.23	0.97 ± 0.10	0.14 ± 0.20
*p* value	0.376	*<0.001*	0.620	*<0.001*	0.985	0.106	0.971	0.178	1.000	0.126

TPR and FDR are averaged across all 36 experiments (see Additional file 2: Table S1 and Additional file 3: Table S2 for details), statistically significant results highlighted in italics

Subsequent results are only reported at the unique count >0 taxon-detection threshold.

### Database for metagenome alignment

Table 3 demonstrates that a reference database constructed of only whole genomes improves accuracy. Overall, results using the custom database had higher TPR and lower FDR than results based on RefSeq.

**Table 3:** Average classification performance by metagenome database used

Database	Bacteria		Bacteria		Fungi		Fungi		Virus	
	Species level		Genus level		Species level		Genus level		First taxon level	
	TPR	FDR	TPR	FDR	TPR	FDR	TPR	FDR	TPR	FDR
RefSeq	0.76 ± 0.12	0.56 ± 0.20	0.83 ± 0.13	0.34 ± 0.19	0.78 ± 0.05	0.79 ± 0.19	0.89 ± 0.08	0.72 ± 0.23	0.95 ± 0.14	0.05 ± 0.07
Custom	0.78 ± 0.12	0.34 ± 0.12	0.84 ± 0.13	0.07 ± 0.07	0.98 ± 0.05	0.45 ± 0.21	0.96 ± 0.08	0.41 ± 0.19	0.99 ± 0.03	0.08 ± 0.10
*p* value	*0.017*	*<0.001*	0.507	*<0.001*	*<0.001*	*<0.001*	*<0.001*	*<0.001*	0.353	0.378

TPR and FDR are averaged across 18 experiments each, statistically significant results highlighted in bold

Subsequent results are reported using only the custom database. The ability of the protocol to classify microbiome samples containing organisms, which are not represented in the reference database, is evaluated below (see Real data analysis).

### Impact of assembler

**Fig. 4:**
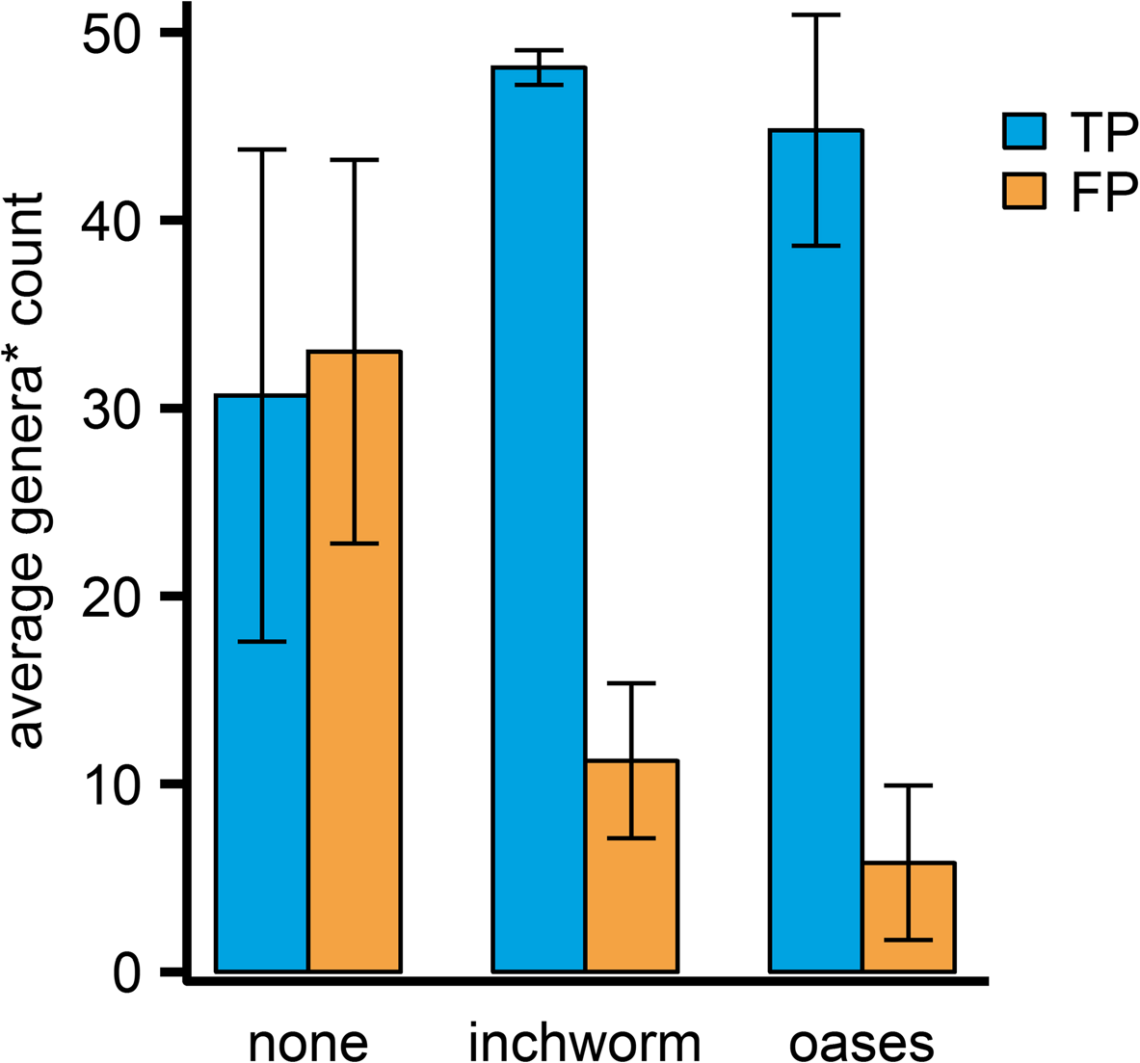
The number of genera identified by IMSA+A using different read assemblers. TP and FP counts are averaged over the nine simulated datasets (Table 1). *Viral genera are counted using the first defined taxon count (see Methods for details)

Two assemblers capable of de novo metatranscriptome sequence assembly were evaluated for inclusion in the metataxonomics protocol. IMSA+A was run on the same nine datasets (Table 1) using the new count method and custom database, varying the assembler used (Fig. 4). The inclusion of an assembler improves taxonomy classification, both increasing true positives and reducing false positives. Oases lowers the number of FPs to about half of FPs by Inchworm.

Table 4 presents a detailed comparison of IMSA+A results by the assembler used. Taxonomy classification based on Inchworm assembly produces higher TPR and FDR than when using Oases. This demonstrates that the error-correcting steps employed by Oases improve the quality of assembled contigs, fewer but longer (Table 5). The assemblers yield ten to five hundred times fewer sequences after assembly, which significantly reduces the time needed to calculate alignments.

**Table 4:** Average classification performance by the assembler used

	Bacteria		Bacteria		Fungi		Fungi		Virus	
	Species level		Genus level		Species level		Genus level		First taxon level	
Assembler	TPR	FDR	TPR	FDR	TPR	FDR	TPR	FDR	TPR	FDR
Inchworm	0.82 ± 0.02	0.40 ± 0.10	0.88 ± 0.03	0.10 ± 0.09	1.00 ± 0.00	0.56 ± 0.11	0.98 ± 0.03	0.52 ± 0.11	1.00 ± 0.00	0.13 ± 0.12
Oases	0.74 ± 0.17	0.28 ± 0.12	0.80 ± 0.17	0.05 ± 0.05	0.96 ± 0.07	0.33 ± 0.23	0.93 ± 0.10	0.30 ± 0.21	0.98 ± 0.04	0.03 ± 0.05
*p* value	*0.010*	*0.010*	*0.005*	*0.022*	0.356	*0.012*	0.365	*0.005*	*0.008*	0.057

TPR and FDR are averaged across 9 experiments each, statistical significant results highlighted in italics

**Table 5:** Measures of assembly characteristics by the assembler program

Assembler	Read length	Number of contigs (thousands)	N50 contig length	Median contig length
Inchworm	50	385.7	68	62
Oases	50	6.5	409	195
Inchworm	100	310.7	315	192
Oases	100	119.3	584	283
Inchworm	150	248.6	689	305
Oases	150	173.4	1047	501

### Other key parameters

Further simulation experiments (Additional file 4: Table S3) investigated such parameters as read length (50, 100, or 150 bases, and a variable read length), mutation rate (0, 1, or 3%), composition and mixture of species, coverage (see Additional file 1 “Key Parameters”).

Only coverage was identified as a critical parameter (Additional file 1: Table S4). If it drops below 1, the protocol shows difficulties in detecting organisms (Additional file 1: Tables S4 and S5). Coverage is determined by read length, sequencing depth, gene expression, and the number of organisms present. The protocol is robust to variation in these individual parameters, as long as the resulting coverage does not go below the critical point (Additional file 1: Tables S5–S7). Classification performance decreases marginally as mutation rate increases up to 3% (Additional file 1: Table S8). Microbiome composition does not affect the protocol performance (Additional file 1: Table S9, Additional file 5: Figure S1, Additional file 6: Figure S2). Additional file 7: Figure S3 demonstrates the cumulative advantage of IMSA+A.

In previous simulation experiments, gene expression and relative abundance were controlled. We repeated the simulation conditions for “50 high” simulation (Table 1) with new randomly selected genomes, varying gene expression from 1 to 1000, and relative abundance from 1 to 20, both in relative units. The results show the protocol performs similarly to the simulation datasets with controlled gene expression and relative abundance (Table 6). Virus classification performance under these conditions shows FDR of 0.18. Thus, with highly variable expression, the protocol may have some difficulties in detecting viruses.

**Table 6:** Classification performance of simulated data set with variable gene and relative abundance by IMSA+A (Oases)

Gene expression and relative abundance	Bacteria		Bacteria		Fungi		Fungi		Virus	
	Species level		Genus level		Species level		Genus level		First taxon level	
	TPR	FDR	TPR	FDR	TPR	FDR	TPR	FDR	TPR	FDR
Fixed^a^	0.74 ± 0.17	0.28 ± 0.12	0.80 ± 0.17	0.05 ± 0.05	0.96 ± 0.07	0.33 ± 0.23	0.93 ± 0.10	0.30 ± 0.21	0.98 ± 0.04	0.03 ± 0.05
Variable	0.77	0.33	0.87	0.04	1.00	0.12	1.00	0.06	0.90	0.18

^a^Average of all previous simulated experiments

### Real data analysis

**Fig. 5:**
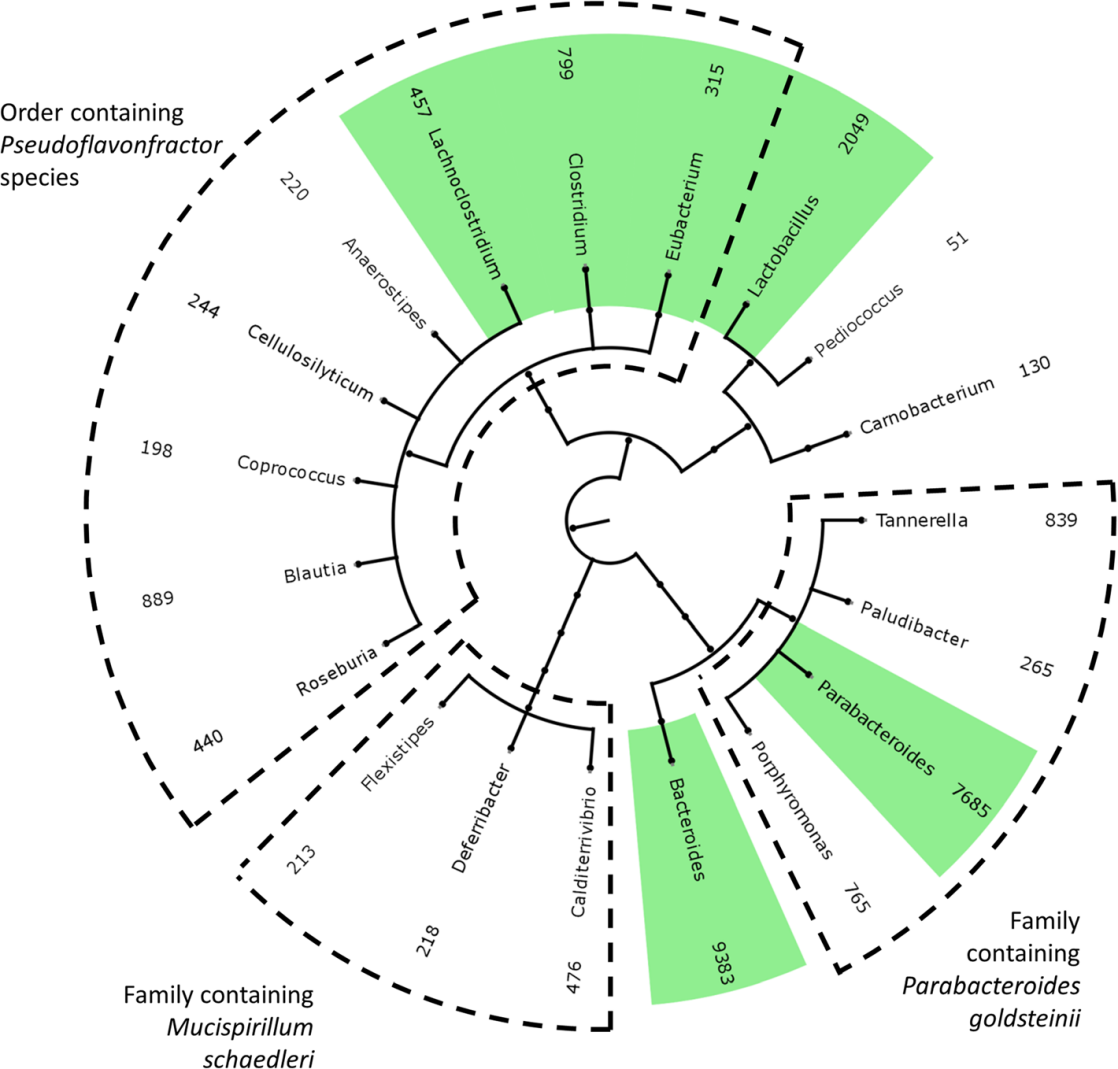
Genera identified by the sum of unique hit counts for all 12 samples. Genera known to be in the samples are *highlighted with a green background*. Groupings of the lowest common ancestors are shown using *sections with dashed lines*

Altered Schaedler Flora (ASF) has long been used as a standardized gut microbiota to colonize germ-free rodents. ASF consists of eight species, *Parabacteroides goldsteinii*, two *Clostridium* species, a *Pseudoflavonifractor* species, *Eubacterium plexicaudatum*, *Mucispirillum schaedleri*, *Lactobacillus murinus*, and *Lactobacillus intenstinalis* [41]. We analyzed RNAseq data derived from the samples taken from the germ-free, ASF colonized mice (NCBI SRA ID: SRA051354) [42, 43] using the IMSA+A (Oases) protocol. Of note, none of the eight species were included in the March 2015 NCBI genomes database used in the IMSA+A protocol. The database does contain other species in the same genera for 6 of the ASF species; namely genera *Parabacteroides*, *Lactobacillus* (2 species), *Clostridium* (2 species), and *Eubacterium*. For species *M. schaedleri*, the lowest common ancestor in the database belonged to family Deferribacteraceae, and for the species of *Pseudoflavonfractor,* the lowest common ancestor belonged to order Clostridiales. Organisms unknown to the database are represented by counting the best homologue; consequently, one unknown organism may be represented by several organisms in the results. To minimize the false positives resulting from the presence of unknown organisms, we treated the 12 mice from the Xiong et. al. study [42] as technical replicates and considered only the genera found in all 12 samples as truly present. There was a total of 380 genera found in any of the 12 mice, of which 19 were found in all mice (Fig. 5). Of these 19, 4 were an exact match for a genus known to be present in ASF; namely *Parabacteroides*, *Lactobacillus*, *Clostridium*, and *Eubacterium*. Additionally, the literature suggests that *Parabacteroides* and *Bacteroides* are the same genera when considering whole genome sequencing data [44], and *Lachnoclostridium* has recently been proposed to account for a subset of *Clostridium* species, cluster XIV, that fall outside of family Clostridiaceae [45]. The *Clostridium* species in ASF are cluster XIV [42], explaining the presence of *Lachnoclostridium* in our results. Of the remaining 13 genera, three belong to family Deferribacteraceae and account for the genus *Mucispirillum* missing in the database. The additional five genera belong to order Clostridiales and likely account for the missing genus *Pseudoflavonfractor*. Three genera are all closely related to genus *Parabacteroides*. The remaining two genera are unrelated to the ASF species.

We compared the output from IMSA+A (Oases) to Kraken and MEGAN CE (MEGAN version 6) (Table 7). MEGAN BLASTN used the same custom database as IMSA+A, allowing for a direct comparison of IMSA+A to MEGAN CE BLASTN. Kraken generates a large number of false positives (55 additional genera). MEGAN CE versions are much more conservative, although still yielding more false positives than the IMSA+A protocol (six and nine by DIAMOND and BLASTN, respectively, vs two by IMSA+A). Moreover, both MEGAN CE versions failed to identify one genus known to be in the samples. The resulting cladograms corresponding to the evaluated methods can be found in Additional file 8: Figure S4; Additional file 9: Figure S5; Additional file 10: Figure S6; Additional file 11: Figure S7.

**Table 7:** Summary of Comparison of Various Tools on ASF data sample

Method	Total genera detected	False positives	True positives	Correct next relative^a^
IMSA+A	19	2	6	11
MEGAN CE DIAMOND	13	6	5	2
MEGAN CE BLASTN	15	9	5	1
Kraken	72	55	6	11

^a^The number of genera representing organisms closely related to the ASF bacteria without sequenced genomes

## Discussion

One of the key challenges for taxonomy classification is handling the ambiguous genomic information. This problem is especially pressing in the case of RNAseq data, where shotgun reads represent more conserved parts of microbial genomes. To address this issue, the IMSA+A protocol includes the following innovations: (1) assembles all RNAseq reads thereby reducing the degree of ambiguity, (2) ignores ambiguous sequences, and (3) uses only high-quality genome assemblies as a reference database.

We recommend using IMSA+A with the Oases assembler based on its lower FDR than Inchworm. However, Inchworm has the advantages of higher TPR and lower variability in overall classification performance. Running the analysis with both assemblers may provide insight to the researcher about coverage. If the Inchworm-based protocol leads to the identification of 50% more organisms than Oases, this may indicate that the sequencing data suffers from low coverage of the microbiome. In theory, any other RNAseq assembler could be used with IMSA+A instead of Oases.

The limited availability of high-quality genomes impedes an exact organism determination in most cases. Obviously, any organism not contained in the reference metagenome database cannot be determined; related organisms will be identified instead as demonstrated in the Real data analysis section of Results. This is a fundamental limitation of any metataxonomics tool.

From the simulated data, IMSA+A consistently has a higher FDR for fungi than for bacteria and viruses (Table 4). Misclassification may be the result of the lower diversity of sequenced fungi: of the few fully sequenced fungi (73 genomes) in the database, many of them are closely related. Another cause of misclassification may be the organization of the taxonomy tree for fungi: closely related organisms are often far apart. For example, *Schizosaccharomyces pombe* and *Saccharomyces cerevisiae* have the lowest common taxon, the phylum Ascomycota, yet their genomes are similar enough to tie top BLAST hits for many queries. The need to revise the fungal taxonomy is a recognized problem, which is being addressed—fungal classifications are revised when genetic evidence is considered [46]. Thus, we hypothesize that the reduction in FDR by classifying organisms at the genus level may help for bacteria but not for fungi, due to the underdeveloped phylogeny of the latter.

IMSA+A has limitations on its applicability. Taxonomy counts are often used to approximate relative abundance of organisms. IMSA+A should not be used for abundance estimation. First, IMSA+A output is counting data for assembled sequences, not the number of identical transcripts. Second, mRNA expression confounds such an analysis, because counts vary by individual gene expression, which depends on multiple intractable factors. IMSA+A also should not be used with DNAseq data. RNA and DNA assembly are disparate problems, whereas Oases is designed for assembly of RNAseq data only.

## Conclusions

We present a new protocol (IMSA+A) to meet the need for metagenomic taxonomy classification from RNAseq data. From the comprehensive evaluation of the protocol, we found the following. De novo assembly of RNAseq data reduces computation time and increases accuracy. The use of only high-quality, complete genomes in the reference database greatly reduces a false positive rate for taxonomy classification. IMSA+A is robust for both short and long sequences, different mutation rates, variable gene expression and relative abundance, and various microbe community compositions without restricting the type of organisms classified. IMSA+A is the first metataxonomics tool for RNAseq without restrictions on organisms in the reference database.

IMSA+A also creates an opportunity to analyze old transcriptome data, which was previously not analyzed for taxonomic content. With the growing appreciation of the microbiome and its functional role in different contexts, such as environment and human health, there is a need for re-analysis of the existing RNAseq datasets, specifically for extracting the microbiome information from the reads previously considered as “garbage” and dismissed as not aligning to a reference genome. Therefore, IMSA+A gives researchers a second use for their metatranscriptome data, as well as a possible way to minimize the cost of experiments.

Simulation experiments demonstrated that low sequencing coverage limits the protocol’s ability to detect organisms, whereas database selection, de novo assembly of shotgun reads, and stricter counting scheme improve classification performance. The analysis of real RNAseq data showed that the protocol is capable of detecting related taxa when the organisms are not in the reference genome database. Moreover, its performance is better than state-of-the-art methods for metataxonomics.

## Additional files

Additional file 1: Key parameters, survey of metataxonomic tools, performance on real data, and simulated gene expression. (DOCX 82 kb)
Additional file 2: Table S1. TPR and FDR are averaged across all 36 experiments. (XLSX 57 kb)
Additional file 3: Table S2. The new counting and original IMSA counting methods. (XLSX 59 kb)
Additional file 4: Table S3. Simulation experiments. (XLSX 65 kb)
Additional file 5: Figure S1. Distributions of microbiome communities generated for simulation experiments in terms of percentage (A) and species counts (B). The sequence percentages not displayed in the chart are human. High coverage—high bacteria coverage treatments (Table S4). Low Coverage—low coverage treatment for all organisms (Table S5). 3.3× bacteria—increased number of bacteria *vs* baseline; 2× fungi—increased number of fungi *vs* baseline; 5× virus—increased number of viruses *vs* baseline (Table S9). Out of 96 scenarios, 84 use the baseline composition. (PNG 53 kb)
Additional file 6: Figure S2. Phylogeny of microbiome communities generated for simulation experiments. Overall, IMSA+A is robust to large changes in composition (Table S9). (PNG 1450 kb)
Additional file 7: Figure S3. Comparison of the average number of genera detected for simulated datasets (Table 1) by the tested metataxonomic tools. The actual number of genera present is 52. IMSA+A was run with Oases assembler and custom database. *Viral genera are counted using the first defined taxon count for IMSA+A (see Methods for details). (PNG 136 kb)
Additional file 8: Figure S4. Genera identified by IMSA+A in all twelve samples containing ASF. Genera highlighted in green match organisms known to be present in the samples. Other colors represent “close relatives” with sequenced genomes to these ASF constituting organisms, which do not have sequenced genomes. Gold represents bacteria in the order Clostridiales, blue represents family Deferribacteraceae, and purple represents the same family as genus *Parabacteroides*. (PNG 337 kb)
Additional file 9: Figure S5. Genera identified by Kraken in all twelve samples containing ASF. Colors have the same notation as in Figure S4. (PNG 585 kb)
Additional file 10: Figure S6. Genera identified by MEGAN CE with DIAMOND in all twelve samples containing ASF. Colors have the same notation as in Figure S4. (PNG 257 kb)

Additional file 11: Figure S7. Genera identified by MEGAN CE with BLASTN in all twelve samples containing ASF. Colors have the same notation as in Figure S4. (PNG 255 kb)

## Abbreviations

16S: Prokaryotic 16S ribosomal RNA gene sequencing
18S: Fungal 18S ribosomal RNA gene sequencing
ASF: Altered Schaedler flora
DNAseq: Shotgun DNA sequencing
FDR: False discovery rate
FPKM: Fragments per kilobase of exon per million reads mapped, a normalized unit for gene expression measurement
ITS: Internal transcribed spacer
LCA: Lowest common ancestor
NCBI: National Center for Biotechnology Information
RNAseq: Ribosomal depleted shotgun RNA sequencing
TPR: True positive rate
