# Supplementary material for: A fast and robust protocol for metataxonomic analysis using RNAseq data
Source: Microbiome. 2017 Jan 19;5:7. doi: 10.1186/s40168-016-0219-5 (PMC5244565; doi:10.1186/s40168-016-0219-5)
Supplement: Additional file 1: — Key parameters, survey of metataxonomic tools, performance on real data, and simulated gene expression. (DOCX 82 kb) [file 40168_2016_219_MOESM1_ESM.docx]

# Supplemental Materials

# **Key Parameters**

## Coverage (Tables S4 and S5)

Bacterial coverage was explored by changing the gene selection and/or the number of simulated sequences. The bacteria species chosen were controlled to be the same between these simulations. Bacteria gene selections were controlled between treatments using the same percent gene selection. The sequences for fungi, viruses, and human were controlled and did not change between these groups. Simulation with 50 bases read length for 1 million bacterial sequences and 30 species at 100% gene selection resulted in 0.25 coverage for bacteria, yielding low TPR (33% for bacteria at the genus level using Oases and custom database). With coverage ≥ 1.0, results became consistent with the rest of experiments.

*Placeholder for Table S4*

Additionally, the effect of simulated human reads was evaluated. The presence of human reads increased FDR for bacteria detection, only when bacteria coverage was < 1 (data not shown). This indicates that human reads not subtracted may hinder classification when there is low coverage of the microbiome.

To extend the consideration of coverage beyond bacteria, low coverage for fungi and viruses were investigated by taking the experiment with 50 read length and bacteria coverage 1.11 and repeating with fungi and virus coverage at approximately 0.50. The results suggest that all organisms will be classified less accurately under low coverage.

*Placeholder for Table S5*

## Read length distribution (Table S6)

Illumina sequencing yields fixed length reads, other sequencers generate a distribution. In this experiment, classification performance based on reads of length 50±0 bases is compared under the same conditions with reads distributions 50±5 and 50±10 bases. The results are mixed, but overall, performance for variable length reads is comparable with the fixed length reads.

*Placeholder for Table S6*

## Read length (Table S7)

A counterintuitive result from the main experiment demonstrated that increase in sequence length leads to higher FDR (Table S1). However, sequence length is connected with coverage. The impact of read length on performance was assessed by varying read length and controlling coverage by adjusting sequencing depth, keeping all other parameters the same. In this experiment, increasing the read length improved TPR for longer reads compared to 50 bases, whereas FDR was negatively impacted by the increased coverage only (Table S7). This suggests that coverage, rather than read length, affects FDR. Therefore, having sufficient coverage is critical (Table S1), but classification performance of our protocol is otherwise not dependent on the read length. This is in contrast to previous methods, where classification performance either dropped as read length decreases [1, 2] or was not evaluated for short reads, being tested with minimum sequence length 100 bases [2-6].

*Placeholder for Table S7*

## Mutation rate (Table S8)

Controlling other variables, this experiment compares 0, 1, and 3% mutation rates. No statistically significant differences are observed between the experiments. However, there is some increase in FDR for fungi and viruses at 3% mutation rate.

*Placeholder for Table S8*

## Composition of the microbiome community (Table S9)

Impact of composition of the organisms present in the sample was evaluated by increasing the number of bacteria, fungi, and virus species selected while controlling coverage by increasing sequencing depth proportionally. Figure S1 summarizes how the microbiome composition varied over all experiments. Cladograms of the varying compositions are in Figure S2.

*Placeholder for Figure S1*

**Figure S1.** Distributions of microbiome communities generated for simulation experiments in terms of percentage (**A**) and species counts (**B**). The sequence percentages not displayed in the chart are human. High Coverage – high bacteria coverage treatments (Table S4). Low Coverage – low coverage treatment for all organisms (Table S5). 3.3x bacteria – increased number of bacteria *vs* baseline; 2x fungi – increased number of fungi *vs* baseline; 5x virus – increased number of viruses *vs* baseline (Table S9). Out of 96 scenarios, 84 use the baseline composition.

*Placeholder for Figure S2*

**Figure S2.** Phylogeny of microbiome communities generated for simulation experiments.

Overall, IMSA+A is robust to large changes in composition (Table S9).

*Placeholder for Table S9*

## Survey of Metataxonomic Tools

## Tools Selected for Survey

The following criteria were used to select DNAseq metataxonomic tools for analysis of RNAseq data: (1) public availability (as of August 2015) and feasible installation on a multicore high-performance cluster; (2) flexibility to use custom databases; (3) capability for multiple organism identification; and (4) utilization of alternative methodologies for taxa identification. In the process of manuscript revision, additional, recently released tools were included in survey, following suggestions by reviewers. Several tools were excluded from testing because they could not use custom databases, could not be tested within our existing cost constraints, or failed to give meaningful results (Table S10). We identified several tools representing different analysis strategies, which could be used readily with custom databases: Kraken [3], IMSA [7], Clinical Pathoscope (v2) [1], MetaGeniE [8], and MEGAN CE [9]. Kraken utilizes a pre-computed lookup table mapping k-mers to LCA taxa. IMSA represents an alignment approach with minimal post-analysis. Kraken and IMSA have the added benefit of being easy to install and use. MetaGeniE uses genome coverage analysis after alignment. Clinical Pathoscope uses Bayesian analysis of alignments for taxa identification. MEGAN CE performs metataxonomics using the LCA, weighted LCA, or projection LCA algorithms.

Clinical Pathoscope and MetaGeniE were not operational with our current computer resources (see Survey Results below). Consequently, we took a deeper look at Kraken and IMSA.

## **Survey Results**

### Technology Barriers

#### Large disk space required

Pathoscope2 generates multiple files during the analysis. When analyzing 70 million reads, temporary files exceeded 400 GB and our system halted. SURPI[10] requires 2TB to install. We did not test SURPI because of the cost barrier it presents for dedicated drive space.

#### Prebuilt database

Dependency on a prebuilt database prevents analysis of many microbiota, such as Protists, Algae, and Fungi. Some tools with prebuilt databases may allow building custom databases (MetaPhlAn2, MetaGeniE), but this is not considered a standard feature and thus is difficult to use. We could not obtain a database to test MetaCV because the repository files with a prebuilt database were corrupted, while the tool provided to build a custom reference database relies on sequence GI numbers, which are no longer supported by NCBI.

#### Poor scaling

MetaGeniE had a high computation time. To process 70 million reads, it was approximately half way complete after 10,000 cpu*hours. It halted when it reached our maximum wall time of 11,520 hours (30 days @ 16 cores).

MEGAN5 is a program deployed for desktops. It cannot scale to large volume. However, MEGAN CE has addressed this problem.

#### Limitations with handling RNAseq

MetaPhlAn2 [10, 11] operates with preselected sets of gene markers that enable distinction of organisms at different clade levels and estimation of relative abundance of a given clade in terms of cell counts. Moreover, since the database catalogs less than 5% of all sequenced microbial genes, it is unlikely that the marker genes will be always expressed and sufficiently abundant to be detected within the metatranscriptome.

### Classification Performance of Kraken and IMSA

Kraken and IMSA with custom and NCBI NT databases, respectively, discovered a large number of genera within 9 simulated datasets (Table 1). Figure S3 compares the average number of genera detected by IMSA, Kraken, and our protocol IMSA+A. On average, IMSA detected 572.7 genera and Kraken found 152.8, when actually only 52 genera were present. The poor signal-to-noise ratio when using RNAseq data makes these methods unsuitable.

*Placeholder for Figure S3*

**Figure S3.** Comparison of the average number of genera detected for simulated datasets (Table 1) by the tested metataxonomic tools. The actual number of genera present is 52. IMSA+A was run with Oases assembler and custom database. *Viral genera are counted using the first defined taxon count for IMSA+A (see Methods for details).

## Methods

### Software Tools

A modified version of IMSA (pIMSA) was used for testing. pIMSA shortens execution time 10-20 fold by utilizing the parallel capabilities of pBLAT [12, 13] and distributing the metagenome BLAST alignment into 36 jobs. IMSA analysis settings were: (1) subtract host (human or mouse) reads with Bowtie2; (2) subtract host reads with BLAT, E-value < 10^-8^; (3) align to metagenome with BLAST, E-value < 10^-8^. The new E-value cutoff, modification from IMSA default 10^-12^, was chosen to increase sensitivity for short (50bp) reads. MEGAN CE was used with default parameters, weighted LCA algorithm, varying only the alignment tool (DIAMOND *vs* BLASTN).

Kraken was modified by adding a postprocessor to tabulate data. Kraken taxa assignments were counted at species and genus level to match IMSA output format. Taxa assigned above species level or above genus level were not included in the species and genus reports, respectively.

Performance of taxonomic classifications was evaluated at the genus level. Genomes of many species remain not sequenced, and consideration of genera enables improved sensitivity in identification of fungi and bacteria. Some sequenced viruses are missing either a species or genus assignment in NCBI taxonomy, therefore viral genus detection is problematic.

### Reference Databases

Metataxonomic tools recommend or require different metagenome databases. Clinical Pathoscope recommends and defaults to NCBI NT database. MetaGeniE requires NCBI RefSeq database. Kraken, IMSA, and MEGAN CE BLASTN allow the use of any metagenome database. MEGAN CE DIAMOND can use any metaproteome database.

Specific versions of the genomic databases used in this work are NCBI NT database as of February 8, 2015 (<ftp://ftp.ncbi.nlm.nih.gov/blast/db/FASTA/nt.gz>), NCBI Genomes database as of March 1, 2015 (.fna files from [ftp://ftp.ncbi.nlm.nih.gov/genomes/](ftp://ftp.ncbi.nlm.nih.gov/genomes/*.fna)), NCBI Genomes (transcriptomes) database as of May 28, 2015 (.ffn files), NCBI RefSeq database version January 2015 (<ftp://ftp.ncbi.nlm.nih.gov/refseq/>). In an attempt to boost speed, truncated versions of the databases NCBI NT (TNTDB) and NCBI Genomes (TNGDB) were also tested, compiled of only bacterial, fungal, and viral sequences. DIAMOND used the NCBI NR database as of October 4, 2016.

For Kraken, a custom database (CSTDB) was constructed by combining the standard Kraken database (generated by its utility) with additional complete genome sequences of microorganisms, sourced from Genbank. CSTDB consists of 19,196 organisms total, including 171 fungi, 3350 bacteria, 15448 viruses, and 227 other (primarily Viridiplantae, Metazoa, protists, and artificial sequences).

## 3. Performance on Real Data

The cladograms below were generated to allow a visual comparison of the results from IMSA+A (Oases), Kraken, MEGAN CE DIAMOND, and MEGAN CE BLASTN.

*Placeholder for Figure S4*

**Figure S4.** Genera identified by IMSA+A in all twelve samples containing ASF. Genera highlighted in green match organisms known to be present in the samples. Other colors represent “close relatives” with sequenced genomes to these ASF constituting organisms, which do not have sequenced genomes. Gold represents bacteria in the order *Clostridiales*, blue represents family *Deferribacteraceae*, and purple represents the same family as genus *Parabacteroides*.

*Placeholder for Figure S5*

**Figure S5.** Genera identified by Kraken in all twelve samples containing ASF. Colors have the same notation as in Figure S4.

*Placeholder for Figure S6*

**Figure S6.** Genera identified by MEGAN CE with DIAMOND in all twelve samples containing ASF. Colors have the same notation as in Figure S4.

*Placeholder for Figure S7*

**Figure S7.** Genera identified by MEGAN CE with BLASTN in all twelve samples containing ASF. Colors have the same notation as in Figure S4.

## 4. Simulated Gene Expression

There is no precedent in the literature for a simulation of metatranscriptome with randomized populations drawn from the entire Metagenome featuring variable gene expression, so we have had to make reasonable accommodation.

While DNAseq Microbiome data sets are typically simulated fully in software, transcriptome data is often simulated using a gene expression matrix derived from real data, because gene expression profiles vary by circumstance, resulting from differences due to biological variability, experimental environment and instrumental settings. A simulation modelled after real data is not possible for this task, because the data availability for simulated organisms is insufficient. Therefore, some simplification is required. We simulated a distribution of gene expression using the formula[14] presented by Flux Simulator[15] (Equation 1).

$y=y_{0}x^{k}e^{-\left( \frac{x}{a}+\frac{x^{2}}{b^{2}} \right)}$ (1)

The default settings for the mammalian fit model were used (k=-0.6, a=9500, b=a^2^), since no universal parameters for the microbiome are defined. We chose y_0_ = 1000, so that choosing a random integer x from 1 to 12000 allows a continuous expression range of 1 to 1000.

References

1. Byrd AL, Perez-Rogers JF, Manimaran S, Castro-Nallar E, Toma I, McCaffrey T, Siegel M, Benson G, Crandall Ka, Johnson WE: **Clinical PathoScope: rapid alignment and filtration for accurate pathogen identification in clinical samples using unassembled sequencing data.** *BMC bioinformatics* 2014, **15**:262.

2. Brady A, Salzberg SL: **Phymm and PhymmBL: metagenomic phylogenetic classification with interpolated Markov models.** *Nature methods* 2009, **6**:673-676.

3. Wood DE, Salzberg SL: **Kraken: ultrafast metagenomic sequence classification using exact alignments.** *Genome biology* 2014, **15**:R46.

4. Kostic A, Ojesina A, Pedamallu C: **PathSeq: software to identify or discover microbes by deep sequencing of human tissue**. *Nature …* 2011, **29**.

5. Freitas TaK, Li P-E, Scholz MB, Chain PSG: **Accurate read-based metagenome characterization using a hierarchical suite of unique signatures**. *Nucleic Acids Research* 2015, **43**:e69-e69.

6. Borozan I, Watt SN, Ferretti V: **Evaluation of alignment algorithms for discovery and identification of pathogens using RNA-Seq.** *PloS one* 2013, **8**:e76935.

7. Dimon MT, Wood HM, Rabbitts PH, Arron ST: **IMSA: integrated metagenomic sequence analysis for identification of exogenous reads in a host genomic background.** *PloS one* 2013, **8**:e64546.

8. Rawat A, Engelthaler DM, Driebe EM, Keim P, Foster JT: **MetaGeniE: Characterizing Human Clinical Samples Using Deep Metagenomic Sequencing.** *PloS one* 2014, **9**:e110915.

9. Huson DH, Beier S, Flade I, Górska A, El-Hadidi M, Mitra S, Ruscheweyh H-J, Tappu R: **MEGAN Community Edition-Interactive Exploration and Analysis of Large-Scale Microbiome Sequencing Data**. *PLoS Comput Biol* 2016, **12**(6):e1004957.

10. Segata N, Waldron L, Ballarini A, Narasimhan V, Jousson O, Huttenhower C: **Metagenomic microbial community profiling using unique clade-specific marker genes**. *Nature Methods* 2012, **9**:811-814.

11. Truong DT, Franzosa Ea, Tickle TL, Scholz M, Weingart G, Pasolli E, Tett A, Huttenhower C, Segata N: **MetaPhlAn2 for enhanced metagenomic taxonomic profiling**. *Nature Methods* 2015, **12**:902-903.

12. Kent WJ: **BLAT---The BLAST-Like Alignment Tool**. *Genome Research* 2002, **12**:656-664.

13. Meng W: **pBLAT**. *unpublished* 2012.

14. **Flux Simulator Gene Expression Profile**. <http://sammeth.net/confluence/display/SIM/4.1.1+-+Gene+Expression+Profile>. Accessed 21 Oct 2016.

15. Griebel T, Zacher B, Ribeca P, Raineri E, Lacroix V, Guigo R, Sammeth M: **Modelling and simulating generic RNA-Seq experiments with the flux simulator**. *Nucleic Acids Res* 2012, **40**(20):10073-10083.

**Table S4.** Classification performance for bacteria at genus level by bacteria coverage.

| **Read** | **Bacteria** | **Inchworm** | | | **Oases** | |
| --- | --- | --- | --- | --- | --- | --- |
| **length** | **coverage** | **TPR** | **FDR** | **TPR** | | **FDR** |
| 50 | 0.25 | 0.87 | 0.04 | 0.33 | | 0.00 |
| 50 | 1.11 | 0.90 | 0.07 | 0.83 | | 0.00 |
| 50 | 4.44 | 0.90 | 0.10 | 0.87 | | 0.04 |
| 100 | 0.87 | 0.83 | 0.00 | 0.83 | | 0.04 |
| 100 | 2.22 | 0.90 | 0.07 | 0.87 | | 0.00 |
| 100 | 8.88 | 0.90 | 0.13 | 0.87 | | 0.10 |
| 150 | 1.30 | 0.83 | 0.04 | 0.83 | | 0.07 |
| 150 | 3.33 | 0.90 | 0.13 | 0.87 | | 0.04 |
| 150 | 13.33 | 0.90 | 0.31 | 0.87 | | 0.13 |

**Table S5.** Classification performance at low coverage for fungi and viruses.

|  |  | **Inchworm** | | | | |  | |  | | |  | |  | | | **Oases** | |  | | |  | |  | | |  | |  |
| --- | --- | --- | --- | --- | --- | --- | --- | --- | --- | --- | --- | --- | --- | --- | --- | --- | --- | --- | --- | --- | --- | --- | --- | --- | --- | --- | --- | --- | --- |
|  |  | **Bacteria** | |  | | | **Fungi** | |  | | | **Virus** | |  | | | **Bacteria** | |  | | | **Fungi** | |  | | | **Virus** | |  |
| **Coverage** | | **Species level** | | | | **Species level** | | | | | **First taxon level** | | | | | **Species level** | | | | | **Species level** | | | | | **First taxon level** | | | |
| **Fungi** | **Virus** | **TPR** | **FDR** | | **TPR** | | | **FDR** | | **TPR** | | | **FDR** | | **TPR** | | | **FDR** | | **TPR** | | | **FDR** | | **TPR** | | | **FDR** | |
| 1.10* | 16.24 | 0.83 | 0.32 | | 1.00 | | | 0.44 | | 1.00 | | | 0.00 | | 0.77 | | | 0.15 | | 0.87 | | | 0.07 | | 1.00 | | | 0.00 | |
| 0.50 | 0.50 | 0.83 | 0.32 | | 1.00 | | | 0.21 | | 0.60 | | | 0.00 | | 0.73 | | | 0.15 | | 0.73 | | | 0.00 | | 0.40 | | | 0.00 | |

* This result is from the main experiment (Table S1), included here for reference.

**Table S6.** Classification performance by variable reads length.

|  | **Inchworm** | | |  |  |  |  | **Oases** |  |  |  |  |  |
| --- | --- | --- | --- | --- | --- | --- | --- | --- | --- | --- | --- | --- | --- |
|  | **Bacteria** | |  | **Fungi** |  | **Viruses** |  | **Bacteria** |  | **Fungi** |  | **Viruses** |  |
| **Read** | **Species level** | | | **Species level** | | **First taxon level** | | **Species level** | | **Species level** | | **First taxon level** | |
| **Length** | **TPR** | **FDR** | | **TPR** | **FDR** | **TPR** | **FDR** | **TPR** | **FDR** | **TPR** | **FDR** | **TPR** | **FDR** |
| 50 ±0* | 0.80 | 0.25 | | 1.00 | 0.44 | 1.00 | 0.00 | 0.30 | 0.18 | 0.87 | 0.00 | 0.90 | 0.00 |
| 50 ±5 | 0.77 | 0.21 | | 1.00 | 0.44 | 1.00 | 0.00 | 0.37 | 0.08 | 0.93 | 0.00 | 0.90 | 0.10 |
| 50 ±10 | 0.77 | 0.23 | | 1.00 | 0.42 | 1.00 | 0.00 | 0.40 | 0.14 | 0.87 | 0.00 | 0.90 | 0.00 |

* This result is from the main experiment (Table S1), included here for reference.

**Table S7.** Classification performance by read length.

|  |  |  |  | **Inchworm** | |  |  |  |  | **Oases** |  |  |  |  |  |
| --- | --- | --- | --- | --- | --- | --- | --- | --- | --- | --- | --- | --- | --- | --- | --- |
|  |  |  |  | **Bacteria** |  | **Fungi** |  | **Virus** |  | **Bacteria** |  | **Fungi** |  | **Virus** |  |
| **Read** | **Coverage** |  |  | **Species level** | | **Species level** | | **First taxon level** | | **Species level** | | **Species level** | | **First taxon level** | |
| **Length** | **Bacteria** | **Fungi** | **Virus** | **TPR** | **FDR** | **TPR** | **FDR** | **TPR** | **FDR** | **TPR** | **FDR** | **TPR** | **FDR** | **TPR** | **FDR** |
| 50* | 0.25 | 1.10 | 16.24 | 0.80 | 0.25 | 1.00 | 0.44 | 1.00 | 0.00 | 0.30 | 0.18 | 0.87 | 0.00 | 0.90 | 0.00 |
| 100 | 0.25 | 1.10 | 16.24 | 0.80 | 0.23 | 1.00 | 0.52 | 1.00 | 0.09 | 0.73 | 0.12 | 1.00 | 0.17 | 1.00 | 0.00 |
| 100* | 0.87 | 2.27 | 106.36 | 0.80 | 0.43 | 1.00 | 0.64 | 1.00 | 0.20 | 0.80 | 0.23 | 1.00 | 0.40 | 1.00 | 0.10 |
| 150 | 0.25 | 1.10 | 16.07 | 0.80 | 0.20 | 1.00 | 0.53 | 1.00 | 0.00 | 0.80 | 0.11 | 1.00 | 0.21 | 1.00 | 0.00 |
| 150* | 1.30 | 3.41 | 159.54 | 0.80 | 0.43 | 1.00 | 0.62 | 1.00 | 0.30 | 0.80 | 0.41 | 1.00 | 0.55 | 1.00 | 0.10 |

* This result is from the main experiment (Table S1), included here for reference.

**Table S8.** Average classification performance by mutation rate.

|  | **Bacteria (species)** | | **Bacteria (genus)** | | **Fungi (species)** | | **Fungi (genus)** | | **Virus** |  |
| --- | --- | --- | --- | --- | --- | --- | --- | --- | --- | --- |
| **Mutation** | **TPR** | **FDR** | **TPR** | **FDR** | **TPR** | **FDR** | **TPR** | **FDR** | **TPR** | **FDR** |
| 0% | 0.77 ±0.16 | 0.34 ±0.10 | 0.84 ±0.15 | 0.04 ±0.05 | 0.93 ±0.00 | 0.51 ±0.18 | 1.00 ±0.00 | 0.43 ±0.17 | 1.00 ±0.00 | 0.02 ±0.04 |
| 1% | 0.76 ±0.18 | 0.30 ±0.11 | 0.83 ±0.16 | 0.04 ±0.04 | 0.92 ±0.03 | 0.50 ±0.18 | 0.99 ±0.03 | 0.42 ±0.18 | 0.92 ±0.13 | 0.05 ±0.09 |
| 3% | 0.73 ±0.23 | 0.28 ±0.05 | 0.81 ±0.23 | 0.04 ±0.05 | 0.93 ±0.00 | 0.38 ±0.19 | 1.00 ±0.00 | 0.30 ±0.19 | 0.98 ±0.04 | 0.25 ±0.26 |
| p-value | 0.741 | 0.557 | 0.986 | 0.994 | 0.368 | 0.220 | 0.368 | 0.277 | 0.283 | 0.109 |

* TPR and FDR are averaged across 6 experiments each (Table S3).

**Table S9.** Classification performance by community composition.

|  |  |  | **Inchworm** | |  |  |  |  | **Oases** |  |  |  |  |  |
| --- | --- | --- | --- | --- | --- | --- | --- | --- | --- | --- | --- | --- | --- | --- |
|  |  |  | **Bacteria** | | **Fungi** |  | **Virus** |  | **Bacteria** | | **Fungi** |  | **Virus** |  |
| **Community** | **Mutation** | **Read** | **Species level** | | **Species level** | | **First taxon level** | | **Species level** | | **Species level** | | **First taxon level** | |
| **composition** | **rate** | **length** | **TPR** | **FDR** | **TPR** | **FDR** | **TPR** | **FDR** | **TPR** | **FDR** | **TPR** | **FDR** | **TPR** | **FDR** |
| baseline* | 1% | 50 | 0.83 | 0.32 | 1.00 | 0.44 | 1.00 | 0.00 | 0.77 | 0.15 | 0.87 | 0.07 | 1.00 | 0.00 |
| 3.3x bacteria | 1% | 50 | 0.80 | 0.44 | 1.00 | 0.38 | 1.00 | 0.00 | 0.69 | 0.18 | 0.80 | 0.00 | 0.90 | 0.00 |
| 2x fungi | 1% | 50 | 0.83 | 0.32 | 1.00 | 0.17 | 1.00 | 0.00 | 0.73 | 0.15 | 0.43 | 0.00 | 1.00 | 0.00 |
| 5x virus | 1% | 50 | 0.83 | 0.32 | 1.00 | 0.44 | 0.92 | 0.02 | 0.77 | 0.18 | 0.87 | 0.00 | 0.98 | 0.02 |
| * This result is from the main experiment (Table S1), included here for reference. Baseline community composition reported in the main text as “50 med” (Table 1) and used here for comparison; other tested conditions were altered from baseline to explore how the number of simulated organisms simulated impacts classification. | | | | | | | | | | | | | | |
|  | |  |  |  |  |  |  |  |  |  |  |  |  |  |

**Table S10.** Survey of metataxonomic tools.*

| **Tool** | **Bacteria** | **Viruses** | **Fungi** | **Database** | **Scalability**^1^ |
| --- | --- | --- | --- | --- | --- |
| Kraken | yes | possible | possible | customizable | yes |
| IMSA | yes | possible | possible | customizable | yes |
| Pathoscope2 | yes | possible | possible | customizable | **large temporary disk space** |
| MetaGeniE | yes | possible | possible | **prebuilt** | **low alignment efficiency** |
| MEGAN5 | yes | yes | yes | customizable | **no** |
| MEGAN CE | yes | yes | yes | customizable | yes |
| MetaCV | yes | **no** | **no** | customizable | not tested |
| GOTTCHA | yes | **no** | **no** | **prebuilt** | not tested |
| MetaPhlAn2 | yes | yes | yes | **prebuilt**^2^ | yes |
| SURPI | yes | yes | yes | all inclusive^3^ | yes, cloud computing scale^4^ |

* Highlighted in bold font are identified difficulties in adaptation to analyzing RNAseq data.

^1^ Ability to handle tens of millions reads

^2^ Can process DNAseq data only

^3^ Provides tools to create an up-to-date, comprehensive database for all microorganisms
^4^ Can be installed locally but requires about 2TB of committed disk space.
