# Supplementary figures and images for: A fast and robust protocol for metataxonomic analysis using RNAseq data
Source: Microbiome. 2017 Jan 19;5:7. doi: 10.1186/s40168-016-0219-5 (PMC5244565; doi:10.1186/s40168-016-0219-5)

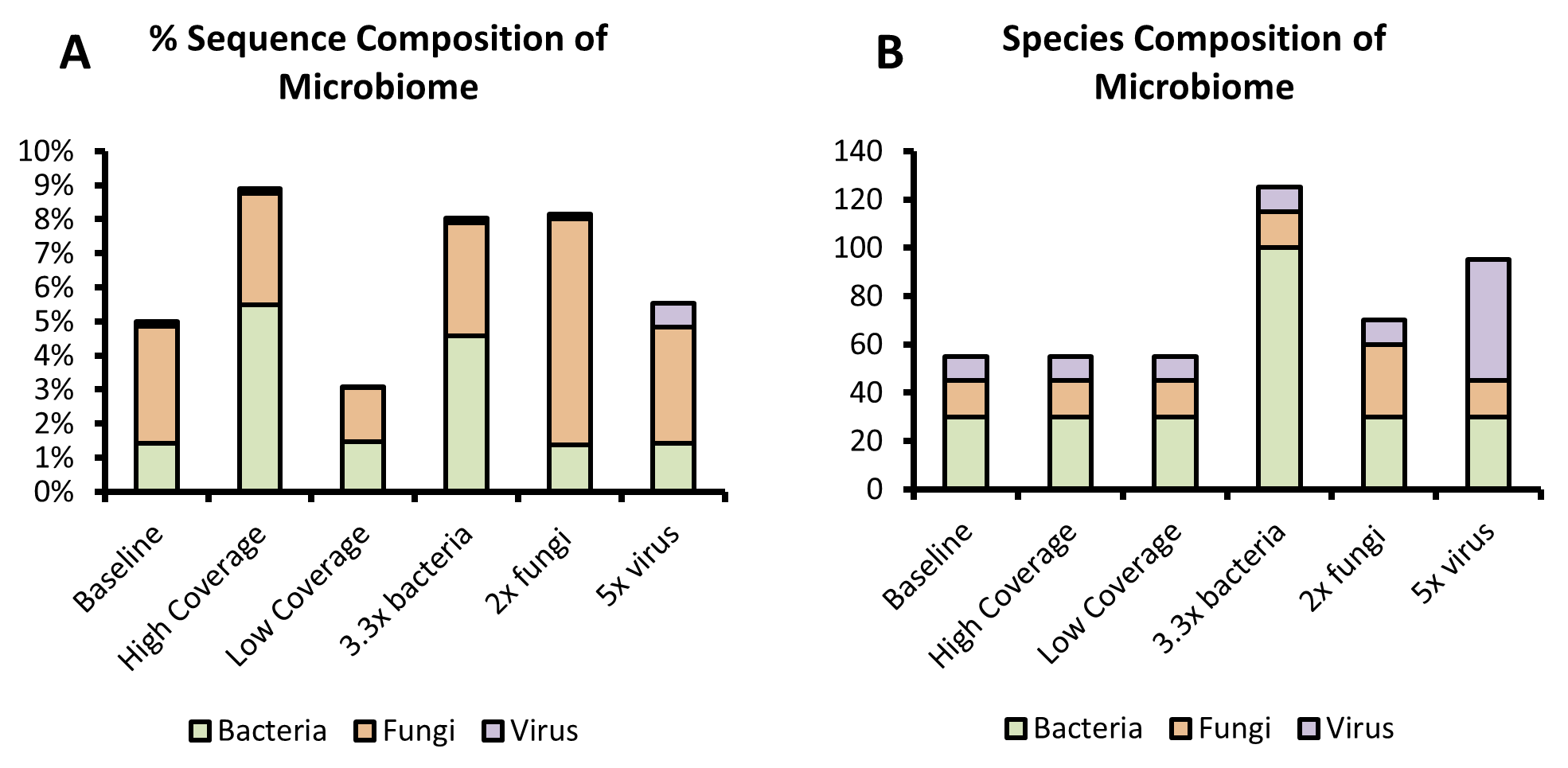

Supplement: Additional file 5: Figure S1. — Distributions of microbiome communities generated for simulation experiments in terms of percentage (A) and species counts (B). The sequence percentages not displayed in the chart are human. High coverage—high bacteria coverage treatments (Table S4). Low Coverage—low coverage treatment for all organisms (Table S5). 3.3× bacteria—increased number of bacteria vs baseline; 2× fungi—increased number of fungi vs baseline; 5× virus—increased number of viruses vs baseline (Table S9). Out of 96 scenarios, 84 use the baseline composition. (PNG 53 kb) [file 40168_2016_219_MOESM5_ESM.png]

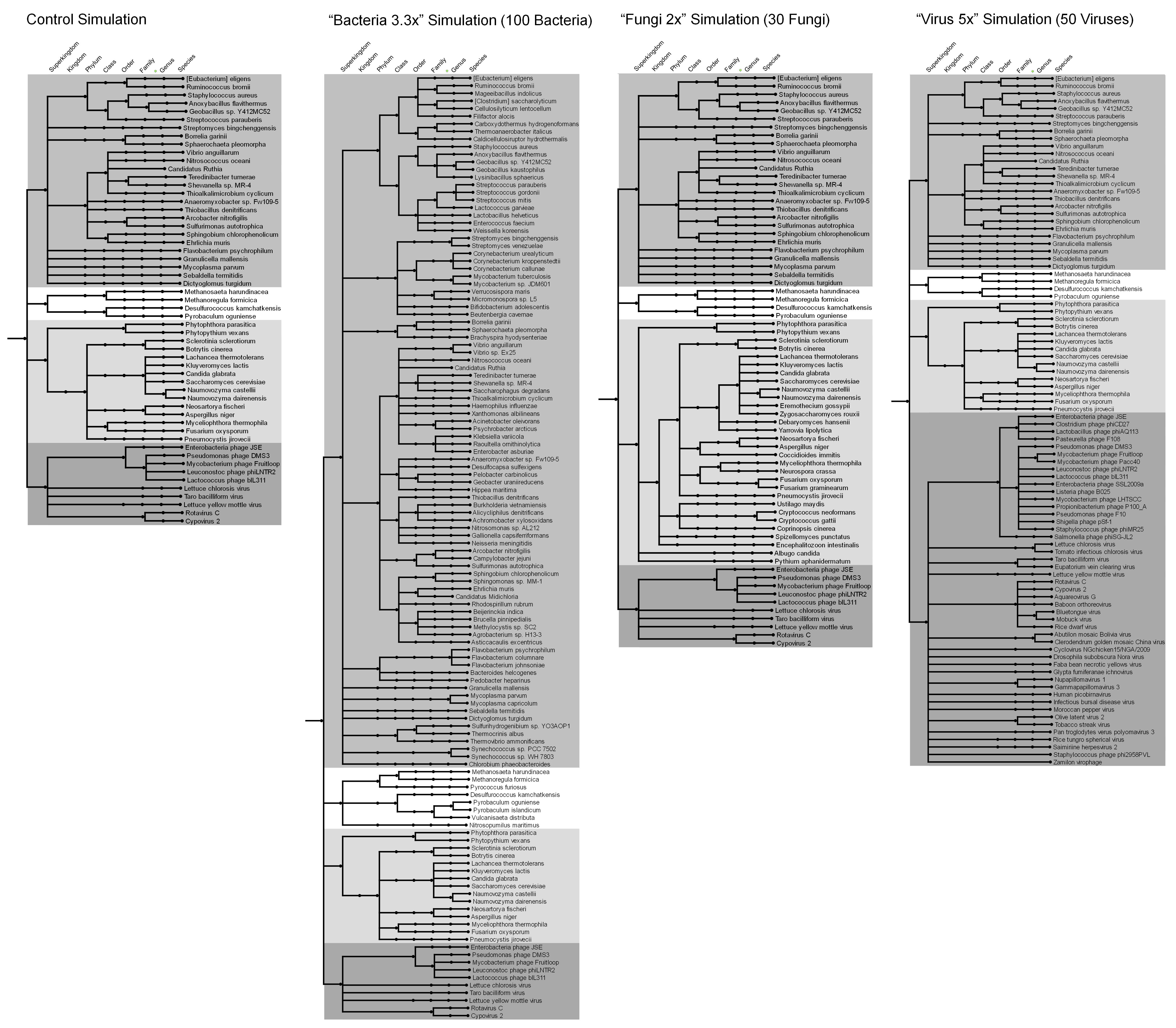

Supplement: Additional file 6: Figure S2. — Phylogeny of microbiome communities generated for simulation experiments. Overall, IMSA+A is robust to large changes in composition (Table S9). (PNG 1450 kb) [file 40168_2016_219_MOESM6_ESM.png]

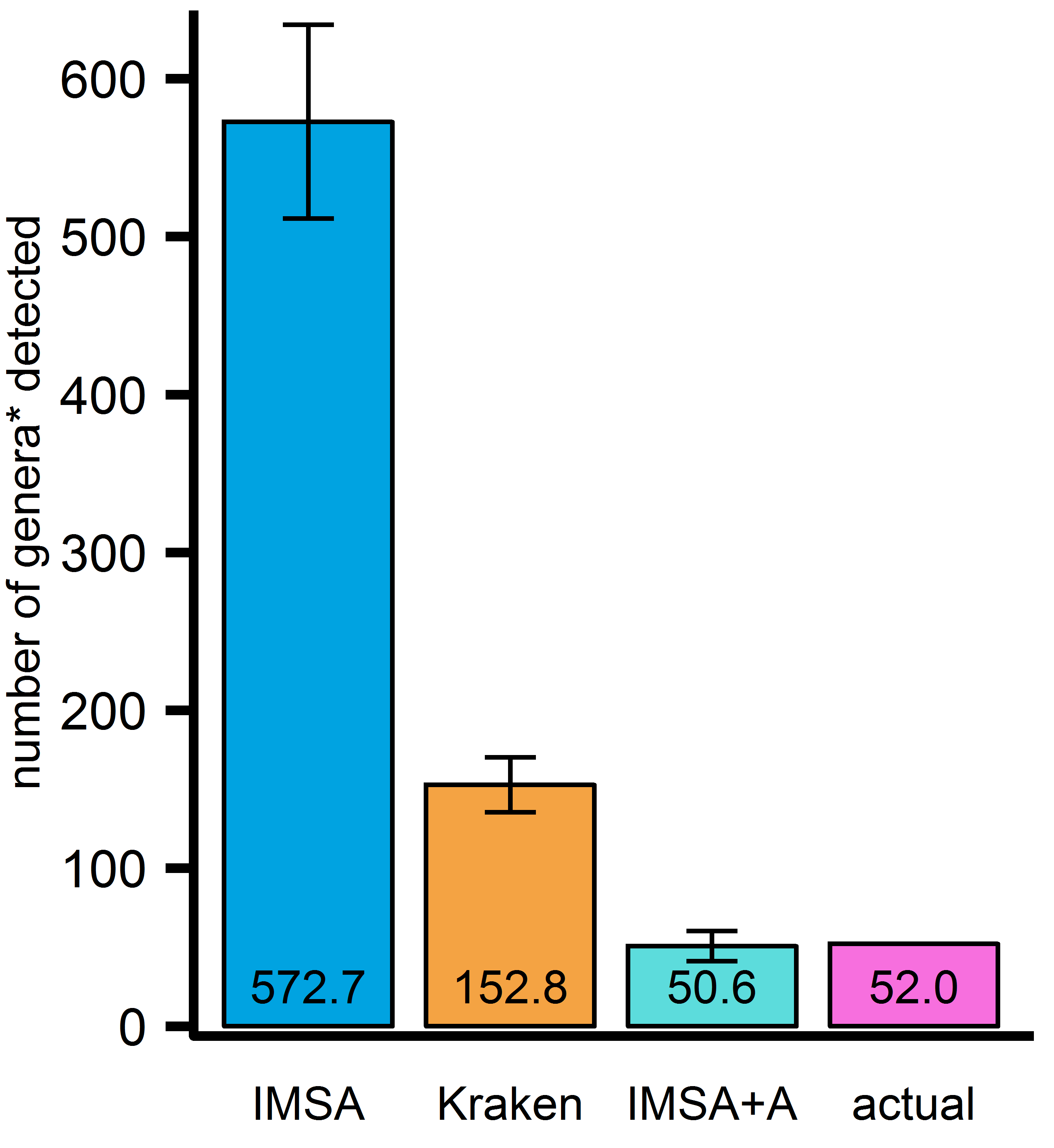

Supplement: Additional file 7: Figure S3. — Comparison of the average number of genera detected for simulated datasets (Table 1) by the tested metataxonomic tools. The actual number of genera present is 52. IMSA+A was run with Oases assembler and custom database. *Viral genera are counted using the first defined taxon count for IMSA+A (see Methods for details). (PNG 136 kb) [file 40168_2016_219_MOESM7_ESM.png]

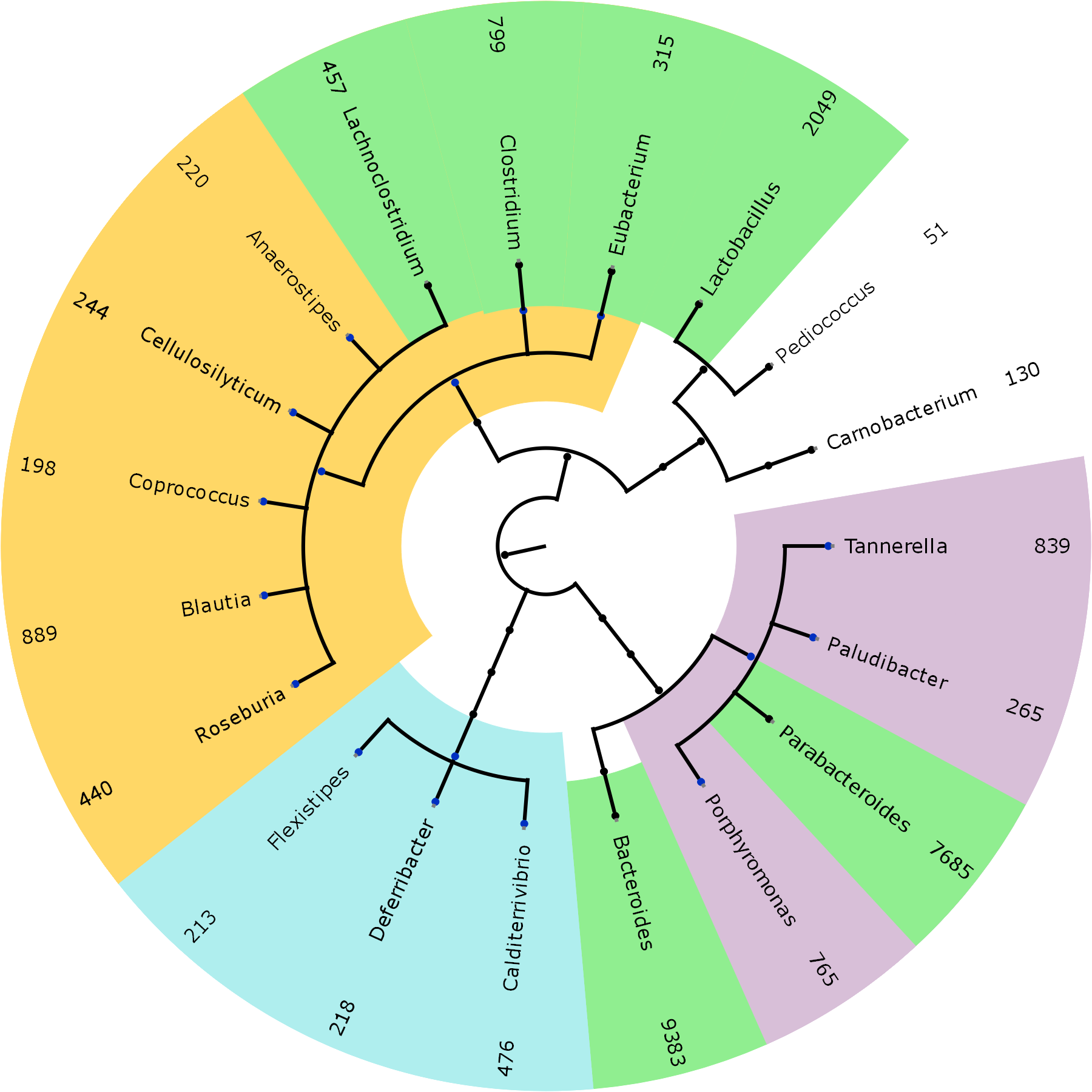

Supplement: Additional file 8: Figure S4. — Genera identified by IMSA+A in all twelve samples containing ASF. Genera highlighted in green match organisms known to be present in the samples. Other colors represent “close relatives” with sequenced genomes to these ASF constituting organisms, which do not have sequenced genomes. Gold represents bacteria in the order Clostridiales, blue represents family Deferribacteraceae, and purple represents the same family as genus Parabacteroides. (PNG 337 kb) [file 40168_2016_219_MOESM8_ESM.png]

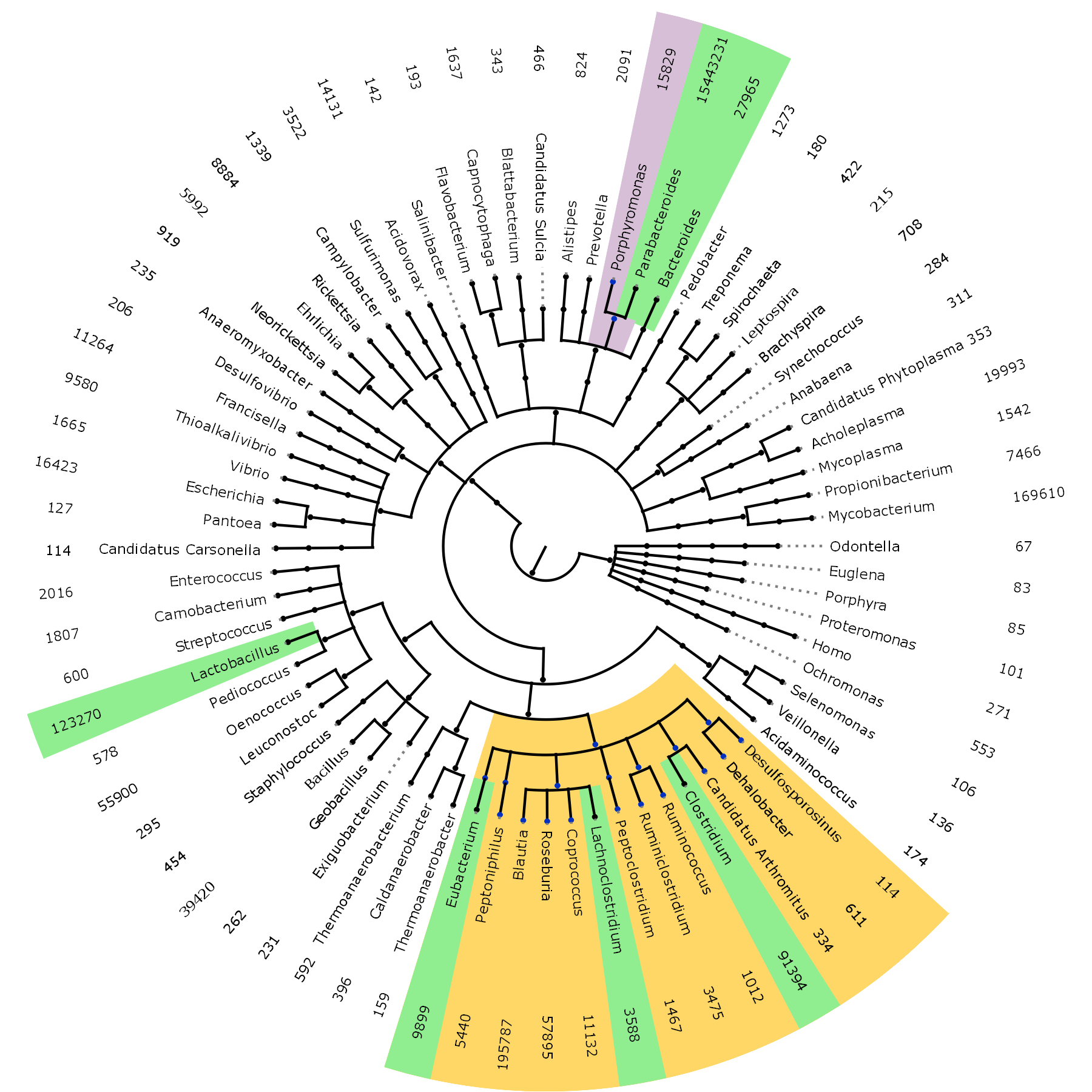

Supplement: Additional file 9: Figure S5. — Genera identified by Kraken in all twelve samples containing ASF. Colors have the same notation as in Figure S4. (PNG 585 kb) [file 40168_2016_219_MOESM9_ESM.png]

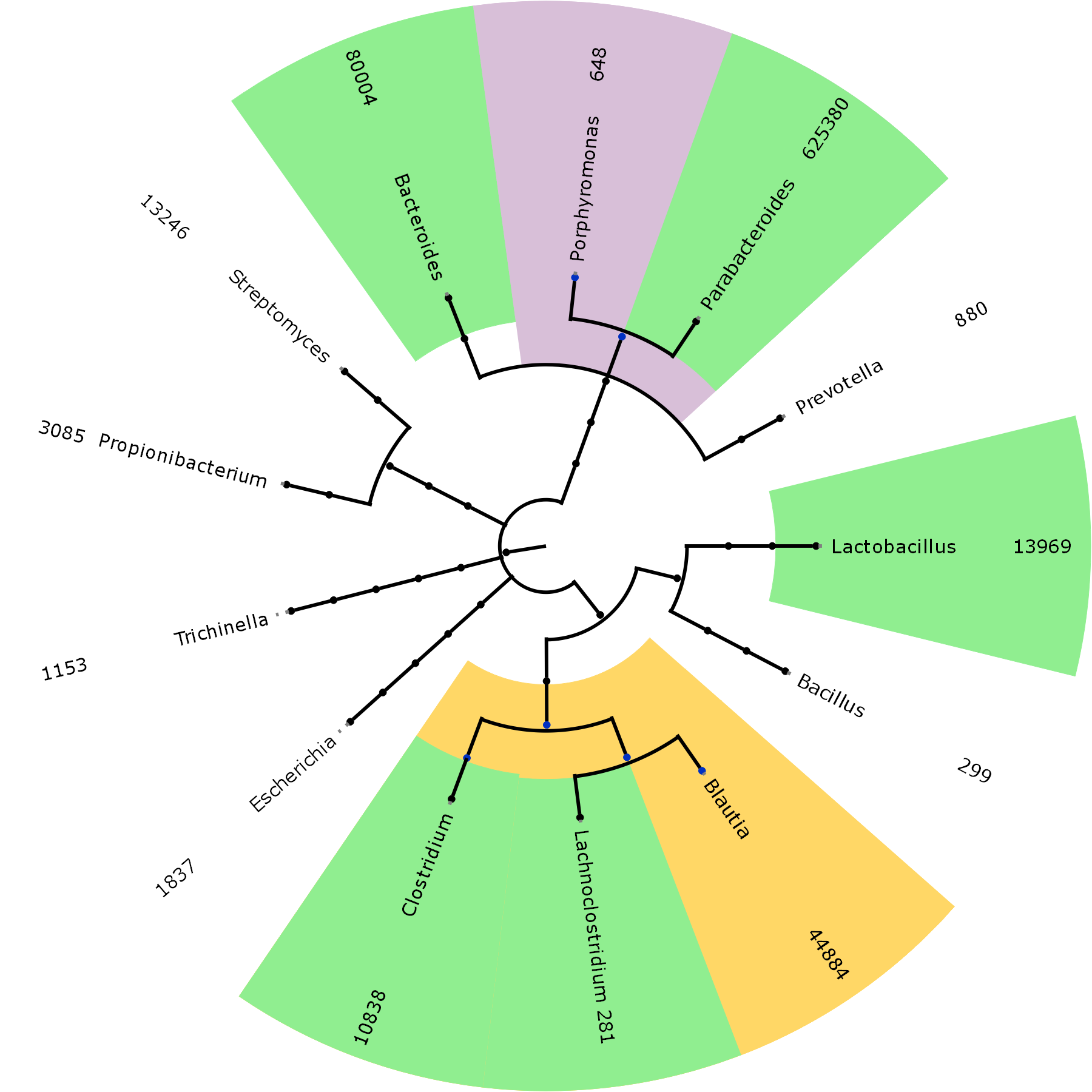

Supplement: Additional file 10: Figure S6. — Genera identified by MEGAN CE with DIAMOND in all twelve samples containing ASF. Colors have the same notation as in Figure S4. (PNG 257 kb) [file 40168_2016_219_MOESM10_ESM.png]

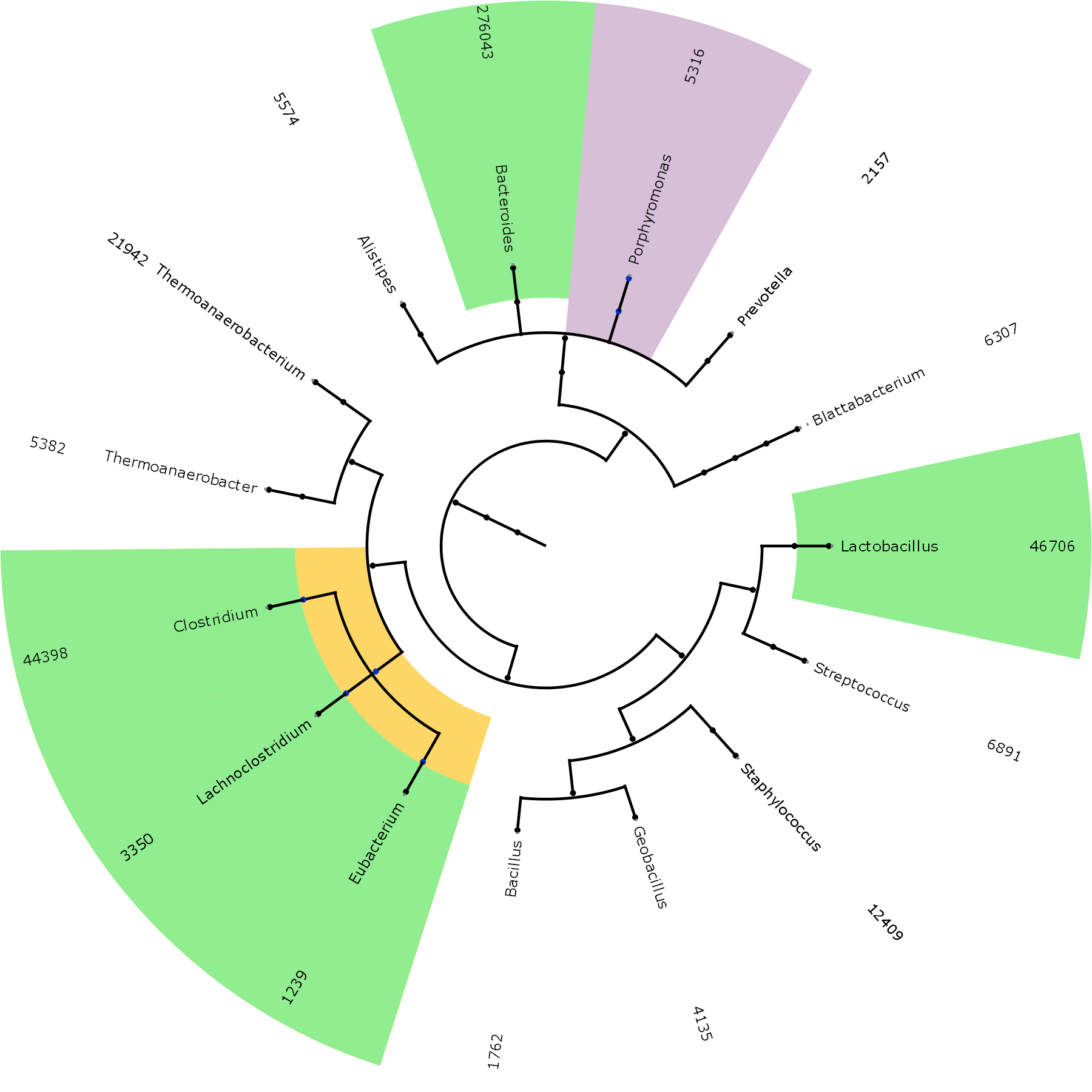

Supplement: Additional file 11: Figure S7. — Genera identified by MEGAN CE with BLASTN in all twelve samples containing ASF. Colors have the same notation as in Figure S4. (PNG 255 kb) [file 40168_2016_219_MOESM11_ESM.png]
